# Stapler versus conventional pharyngeal repair after total laryngectomy: a randomized clinical trial

**DOI:** 10.1007/s00405-024-08696-9

**Published:** 2024-05-13

**Authors:** Elsaeed Ahmed Mandor, Hisham Atef Ebada, Ahmed Musaad Abd El-Fattah, Elsharawy Kamal, Hemmat Baz, Ali Tawfik

**Affiliations:** 1 Department of Otorhinolaryngology, Mansoura University, Mansoura, 35511 Egypt; 2 Phoniatrics, Mansoura University, Mansoura, Egypt

**Keywords:** Laryngeal carcinoma, Total laryngectomy, Pharyngeal closure, Stapler, Pharyngocutaneous fistula, Survival

## Abstract

**Objectives:**

The aim of the current study was to evaluate the functional outcomes of stapler pharyngeal closure after total laryngectomy by the incidence of PCT and assessment of swallowing after surgery. In addition, the study aimed to evaluate the oncological outcomes in terms of patients’ survival rates.

**Methods:**

This randomized clinical trial was conducted on 58 patients with advanced laryngeal carcinoma who underwent total laryngectomy. Patients were randomly assigned to two groups according to the method of pharyngeal repair after laryngectomy: manual closure group (n = 28), and stapler group (n = 30). Functional and oncological outcomes were assessed and compared.

**Results:**

The incidence of pharyngocutaneous fistula was significantly less in the stapler group. Additionally, operative time was significantly shorter and swallowing function was better in the stapler group compared to the manual group. There was no statistically significant difference between groups regarding survival rates.

**Conclusion:**

The stapler is a reliable method for pharyngeal closure after total laryngectomy if the limits of its indications regarding the primary tumor are considered. Stapler closure decreases the incidence of PCF and decreases the surgical time. Good swallowing outcomes are achieved without compromising the oncological outcomes.

## Introduction

Pharyngocutaneous fistula (PCF) is the commonest complication after total laryngectomy [1]. It develops as a result of leakage of saliva through the pharyngeal repair line [2, 3]. PCF contributes to increased morbidity following laryngectomy due to prolonged hospital stay, increased treatment cost, and delayed adjuvant therapy [1, 4, 5]. Moreover, it can potentially lead to mortality secondary to carotid artery blow out or aspiration pneumonia [4, 6]. Research is ongoing to decrease the incidence of PCF [2, 7].

The technique of pharyngeal closure after total laryngectomy plays a critical role in the development of PCF [6]. Adequate repair should be tension-free and watertight to prevent leakage [8]. Pharyngeal repair is typically done by manual suturing which require meticulous surgical technique [9]. Application of staplers for pharyngeal repair was described in 1969, during the resection of the diverticulum of Zenker. However, its first use in total laryngectomy was in 1971 [10]. In contrast to gastrointestinal surgery, stapler pharyngeal closure after total laryngectomy is not widely practiced [11].

Mechanical closure by stapling is reliable and precise as it applies two parallel rows of evenly placed staples on the repaired mucosa [12]. The material of the staples is well tolerated by the tissues with minimal inflammatory reactions and surgical trauma. Good healing is therefore achieved [11].

The aim of the current study was to evaluate the functional outcomes of stapler pharyngeal repair after total laryngectomy by the incidence of PCT and assessment of swallowing after surgery. In addition, the study aimed to evaluate the oncological outcomes in terms of patients’ survival rates.

## Patients and methods

This parallel randomized controlled clinical trial included 58 patients with advanced laryngeal carcinoma (T3 and T4) who underwent total laryngectomy in the Otorhinolaryngology Department, Mansoura University Hospitals, Egypt, during 1 year (February 2021–February 2022).

Informed consents were obtained from all patients. The study was approved by the Mansoura Faculty of Medicine Institutional Research Board (MFM-IRB: MD.21.06.491). This randomized clinical trial was registered at ClinicalTrials.gov (NCT06256263).

**Fig. 1:**
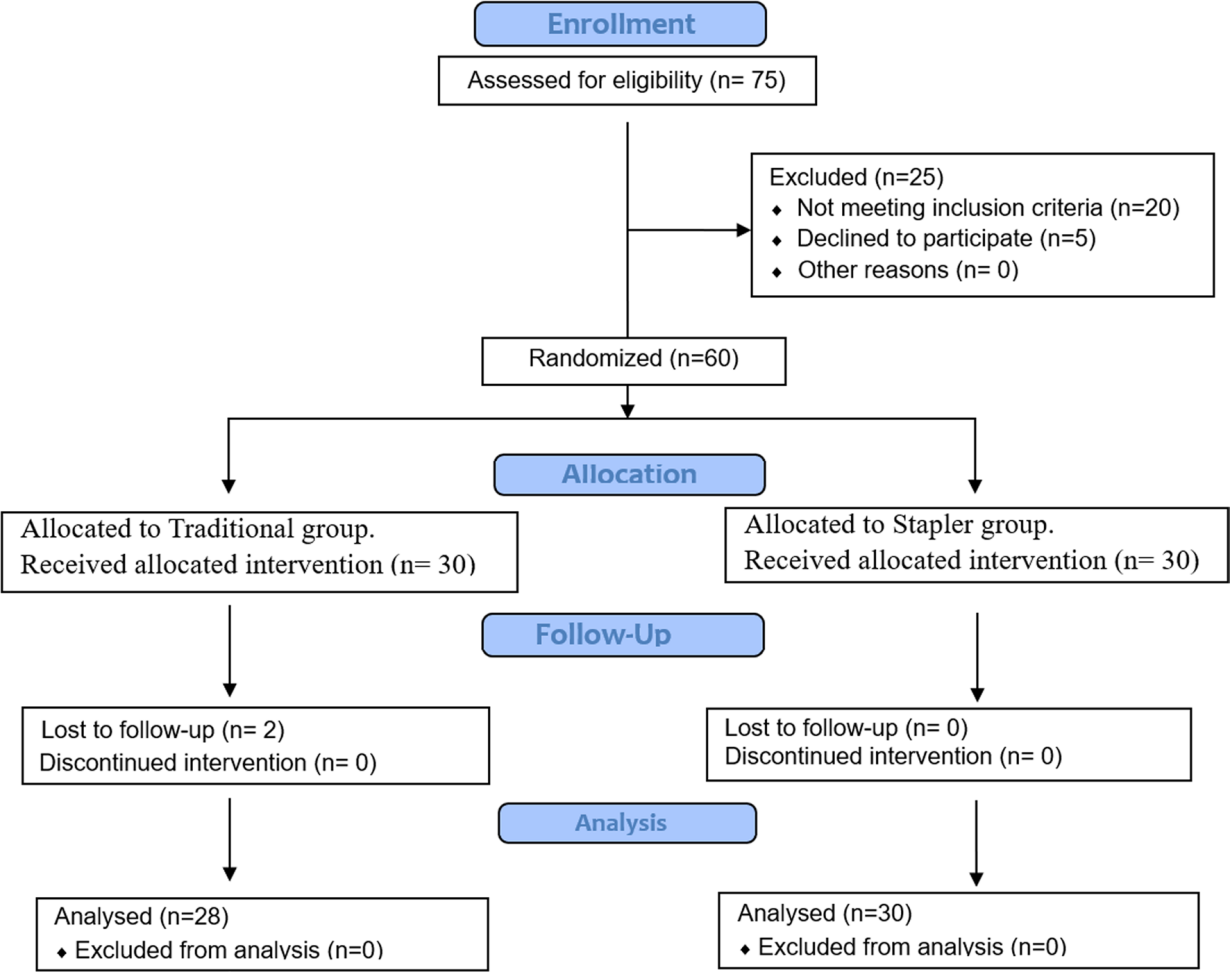
Consort flow chart

All patients who underwent total laryngectomy during the 1 year of the study (n = 75) were eligible for inclusion (Fig. 1). Twenty-five patients were excluded due to tumor extension to the hypopharynx (n = 11) or the tongue base (n = 3), positive surgical margins (n = 2) and patients who declined to participate (n = 9). The remaining 60 patients were analyzed.

The study population (n = 60) were randomly assigned to two groups: stapler group (n = 30), and manual group (n = 30). Randomization was performed based on a computer-generated list of random numbers. Two patients in the manual group were lost to follow up, and consequently the remaining 28 patients were included in the final analysis.

Total laryngectomy was performed for all patients by the same standardized technique by the same surgical team (the authors of this work). Direct laryngoscopy was performed for all patients before laryngectomy to accurately assess the tumor extensions, and to exclude tumors with hypopharyngeal or tongue base extensions.

In the manual group, pharyngeal repair was performed by stitching in two layers; pharyngeal mucosa was sutured by a running inverting technique with 3/0 Vicryl sutures. A second layer was performed to reinforce the mucosal repair by suturing the constrictor muscles. A constrictor myotomy was then performed to decrease the incidence of postoperative dysphagia and difficulties in esophageal speech.

**Fig. 2:**
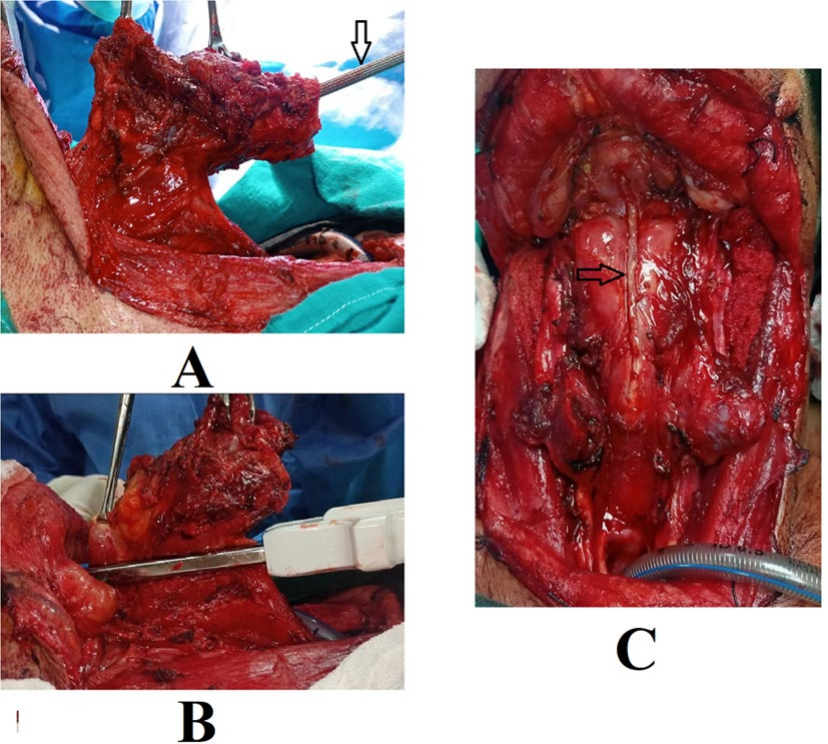
Stapler closure. **A** The larynx is pulled up. A hook (arrow) is introduced through the trachea to hitch the epiglottis. **B** The linear stapler is applied. **C** The pharynx after stapler closure. The arrow points to the line of closure

On the other hand, in the stapler group, a linear stapler was used for pharyngeal closure. To facilitate application of the stapler, the upper cornus of the thyroid cartilage as well as the greater cornus of the hyoid bone were divided and removed. The larynx was then pulled up and elevated by using Allis clamps. A 60-mm linear stapler (Covidien, GIA, with Tri-Staple Technology) was applied longitudinally parallel to the esophagus and pharynx, and close to the under surface of the larynx. The stapler was then activated resulting in closure of both the pharynx and larynx with two rows of staples, and in the same time separation of the pharynx from the larynx (Fig. 2).

In order to prevent the epiglottis free border from being caught between the blades of the stapler, two techniques were applied. The closed technique entailed pulling the epiglottis free border into the endolarynx. A single hook or an Allis clamp was introduced through the lower cut of the trachea up to the epiglottis to grasp and pull it into the laryngeal lumen away from the stapler suture line. Epiglottis is identified by external palpation through the intact vallecular mucosa or guided by inserting an endoscope from the lower tracheal cut. The closed technique was applied in 24 out of 30 patients.

On the other hand, the semi-closed technique was applied in six patients when the laryngeal tumor was very large and totally obstructing the laryngeal lumen making it difficult to insert the hook. In this technique, a small pharyngotomy opening was performed in the mucosa of the vallecula, through which the epiglottis was delivered and grasped. The stapler was then applied and activated while keeping the edges of the pharyngotomy above the blades to achieve complete closure of the pharynx below.

In the stapler group (n = 30), only a single layer closure was performed. The constrictor muscle was not sutured over the mucosal layer.

The primary outcome of this randomized clinical trial was the development of PCF. Secondary outcomes were operative time of pharyngeal repair, postoperative swallowing function and oncological outcomes. Postoperative swallowing was evaluated both objectively and subjectively, 3 months after surgery.

Subjective evaluation was done by application of the Eating Assessment Tool (EAT-10) questionnaire (Table 1). The Arabic version [13] was applied as all the study population were Arabic speaking. EAT-10 is a questionnaire that consists of ten questions, the answers to which are scaled from 0 to 4, where 0 corresponds to no problem and 4 to a severe problem. The total score is calculated by summing individual scores. A score > 3 indicates abnormality. Higher scores indicate more severe swallowing impairment.

**Table 1:** Layout of the component questions of the EAT-10 questionnaire

Circle the appropriate response	0 = No problem 4 = Severe problem				
1. My swallowing problem has caused me to lose weight	0	1	2	3	4
2. My swallowing problem interferes with my ability to go out for meals	0	1	2	3	4
3. Swallowing liquids takes extra effort	0	1	2	3	4
4. Swallowing solids takes extra effort	0	1	2	3	4
5. Swallowing pills takes extra effort	0	1	2	3	4
6. Swallowing is painful	0	1	2	3	4
7. The pleasure of eating is affected by my swallowing	0	1	2	3	4
8. When I swallow food sticks in my throat	0	1	2	3	4
9. I cough when I eat	0	1	2	3	4
10. Swallowing is stressful	0	1	2	3	4
**Total EAT-10:**					

Objective evaluation of swallowing was done by performing video fluoroscopy (VFS). The patients were instructed to swallow liquid (water and barium at a 1:1 ratio) and solid substances mixed to barium. Five ml. (spoon) and 20 ml. (cup) of liquid were given in a continuous swallowing. For solids, the patients were instructed to chew the cookie before swallowing. Residue, strictures, diverticula, as well as cricopharyngeal spasm were documented.

Oncological outcomes were evaluated by assessment of the overall and disease specific survival rates. Patients were considered as being alive with and without oncologic disease; dead with local, regional, or distant disease; dead without oncologic disease. The cutoff point for statistical analysis was February 2024, encompassing a minimum FU of 24 months. Overall survival and disease specific survival curves were calculated using the Kaplan–Meier method, and statistical significance was determined by the Log-Rank test.

Data analysis was performed by the SPSS software, version 25 (SPSS Inc., PASW statistics for windows version 25. Chicago: SPSS Inc.). Qualitative data were described using numbers and percentages. Quantitative data were described using mean ± Standard deviation for normally distributed data after testing normality using Kolmogrov-Smirnov test. The significance of the obtained results was judged at the (≤ 0.05) level. Chi-Square, Fisher exact tests were used to compare qualitative data between groups as appropriate. Student T test was used to compare two independent groups for normally distributed data.

## Results

Fifty-eight participants were included in the current study. Table 2 shows the age and medical comorbidities among the participants. There was no statistically significant difference between groups regarding age and comorbidities. All the participants were males (n = 58).

**Table 2:** Age and comorbidities among the study population

Age and comorbidities	Manual group N = 28(%)	Stapler group N = 30(%)	Test of significance
Age/years Mean ± SD	58.61 ± 5.74	60.53 ± 9.43	t = 0.931 p = 0.356
Pre-operative radiotherapy	6(21.4%%)	7(23.3%)	χ^2^ = 0.03 P = 0.862
Diabetes Miletus	10(35.7%)	9(30.0%)	χ^2^ = 0.215 P = 0.643
Hypertension	18(64.3%)	22(73.3%)	χ^2^ = 0.554 P = 0.457
Liver cirrhosis	0	1(3.3%)	χ^2FET^ = 0.950 P = 1.0
Malnutrition	0	1(3.3%)	χ^2FET^ = 0.950 P = 1.0

*t* student t test, *χ*^*2*^ chi-square test, *FET* fisher exact test

Table 3 shows the tumor characteristics (T stage, detailed tumor extensions to laryngeal subsites, and N stage) and operative data (preoperative tracheostomy, and neck dissection). There was no statistically significant difference between groups regarding the above-mentioned parameters.

**Table 3:** Tumor characteristics and surgical details among the study population

	Manual group N = 28	Stapler group N = 30	Test of significance
T stage			
T3	8 (28.6%)	5 (16.7%)	χ^2^ = 1.18 P = 0.277
T4	20 (71.4%)	25 (83.3%)	χ^2^ = 1.18 P = 0.277
Detailed tumor extensions in the different laryngeal subsites			
Epiglottis	8 (28.6%)	5 (16.7%)	χ^2^ = 1.18 P = 0.277
Aryepiglottic fold	14 (50.0%)	10 (33.3%)	χ^2^ = 1.66 P = 0.198
False vocal cords	16 (57.1%)	15 (50.0%)	χ^2^ = 0.297 P = 0.586
Ventricles	20 (71.4%)	24 (80.0%)	χ^2^ = 0.581 P = 0.446
True vocal folds	26 (92.9%)	30 (100%)	χ^2^ = 2.22 P = 0.229
Anterior commissure	18 (64.3%)	20 (66.7%)	χ^2^ = 0.036 P = 0.849
Arytenoids	20 (71.4%)	25 (83.3%)	χ^2^ = 1.18 P = 0.277
Subglottis	20 (71.4%)	25 (83.3%)	χ^2^ = 1.18 P = 0.277
Pre-epiglottic space	10 (35.7%)	5 (16.7%)	χ^2^ = 2.74 P = 0.098
Paraglottic space	20 (71.4%)	24 (80.0%)	χ^2^ = 0.581 P = 0.446
Thyroid cartilage	20 (71.4%)	21 (70.0%)	χ^2^ = 0.014 P = 0.905
Cricoid cartilage	12 (42.9%)	20 (66.7%)	χ^2^ = 3.32 P = 0.07
N stage			
N0	10 (35.7%)	15 (50.0%)	χ^2^ = 1.21 P = 0.272
N1	10 (35.7%)	9 (30.0%)	χ^2^ = 0.215 P = 0.643
N2	8 (28.6%)	6 (20.0%)	χ^2^ = 0.215 P = 0.643
Preoperative tracheostomy	8 (28.6%)	5 (16.7%)	χ^2^ = 1.18 P = 0.277
Pre-operative radiotherapy	6 (21.4%)	7 (23.3%)	χ^2^ = 0.03 P = 0.862
Neck dissection			
Unilateral	22 (78.6%)	26 (86.7%)	χ^2^ = 0.665 P = 0.415
Bilateral	6 (21.4%)	4 (13.3%)	χ^2^ = 1.19 P = 0.273

*χ*^*2*^ chi-square test

Regarding the primary outcome in the current study, pharyngocutaneous fistula was reported in 6/28 patients in the manual group (21.4%), and in only 1/30 patients in the stapler group (3.3%), with a statistically significant difference (p = 0.035) (Table 4). Spontaneous fistula healing was reported in 4/6 patients in the manual group as well as in the one patient in the stapler group. Two patients in the control group required surgical closure of the fistula after failure of conservative treatment for 3 months.

**Table 4:** Study outcomes

	Manual group N = 28	Stapler group N = 30	Test of significance	Mean difference (95%CI)
Pharyngocutaneous fistula	6 (21.4%)	1 (3.3%)	χ^2FET^ = 4.47 P = 0.035*	
Time of pharyngeal repair/minutes (mean ± SD)	21.07 ± 4.82	1.71 ± 1.04	t = 20.76 p < 0.001*	19.35 (17.48–21.23)
EAT-10 questionnaire	13.8 ± 3	9.8 ± 1.8	t = 6.12 p < 0.001*	3.98 (2.67–5.28)
Video fluoroscopy				
Residue	19 (67.9%)	9 (30.0%)	χ^2^ = 8.31 P = 0.004*	
Strictures	4 (14.3%%)	2 (6.7%%)	χ^2^ = 2.74 P = 0.098	
Diverticula	6(21.4%)	7(23.3%)	χ^2^ = 0.03 P = 0.862	
Cricopharyngeal spasm	6(21.4%)	1(3.3%)	χ^2FET^ = 4.47 P = 0.035*	

*t* student t test, *FET* fisher exact test, *χ*^*2*^ Chi-Square test

*Statistically significant

Secondary outcomes in the current study are shown in Table 4. The time of pharyngeal repair was significantly shorter in the stapler group as the mean time was 1.7 min, while it was 21 min in the manual group (p < 0.001).

Swallowing was also significantly better in the stapler group both subjectively and objectively. The mean EAT-10 score in the manual group was 13.8, while it was 9.8 in the stapler group denoting statistically significant better swallowing scores in the stapler group (p < 0.001). Additionally, the incidence of food residue, strictures and cricopharyngeal spasm in the video fluoroscopy were higher in the manual group than the stapler group.

**Fig. 3:**
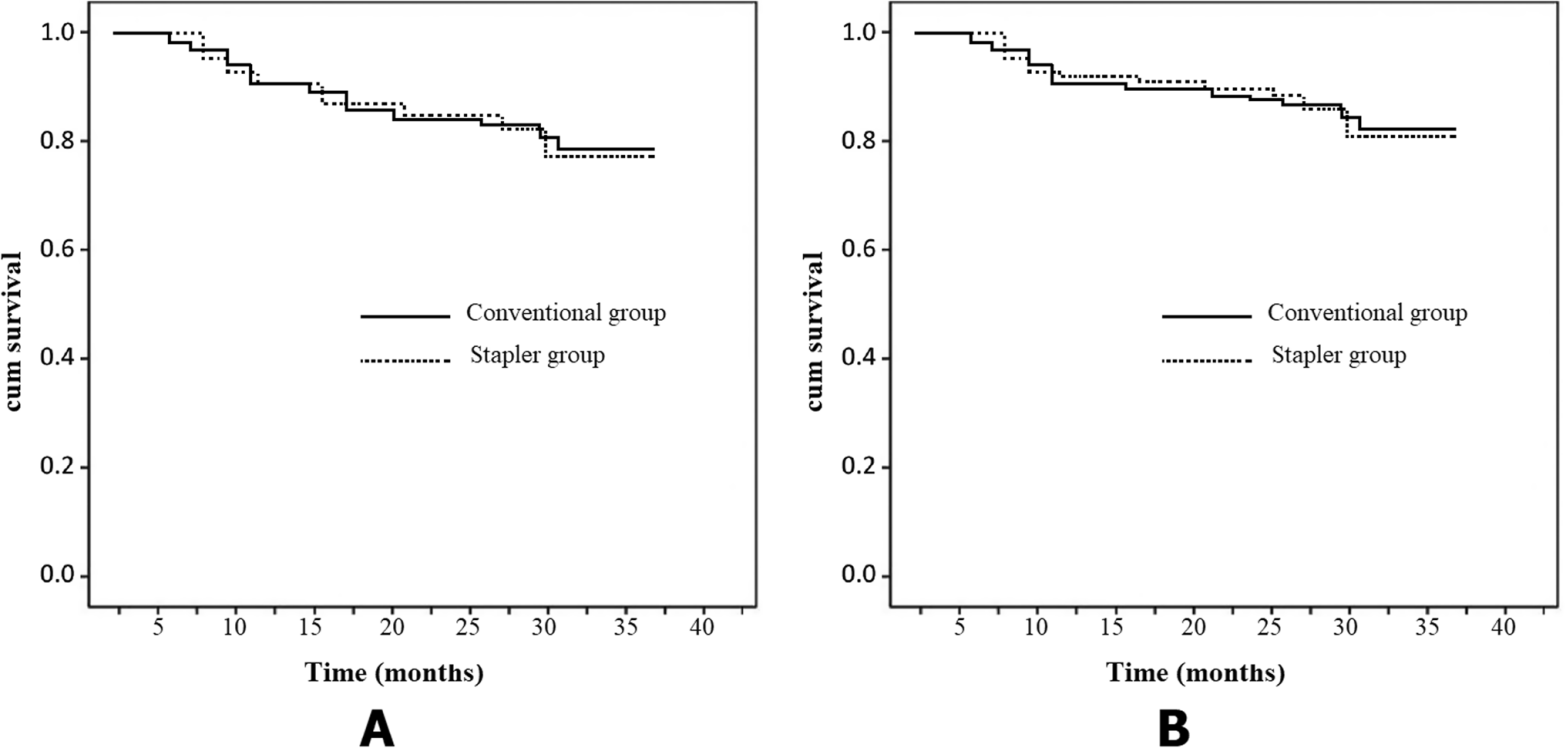
Kaplan–Meier curves of 3-year survival rates. **A** Overall survival rates. **B** Disease specific survival rates

Regarding survival outcomes, the 3-year overall survival was 79.3%, and the 3-year disease specific survival was 81%. The method of pharyngeal repair in the current study did not have a significant impact on the survival rates. Figure 3 show the overall survival and disease specific survival curves, respectively. *P* values were 0.355 and 0.378, respectively, with no statistically significant differences between the groups.

## Discussion

Very few randomized clinical trials exist in the literature comparing between manual and stapler pharyngeal closure after total laryngectomy [12, 14, 15]. The previous clinical trials evaluated the short-term outcomes such as incidence of pharyngocutaneous fistula, operative time, cost, and hospital stay. To the best of our knowledge this is the first randomized clinical trial to evaluate the abovementioned short-term outcomes as well as the long-term oncological outcomes in terms of overall and disease specific survival rates.

Pharyngeal repair techniques appear to have an impact the development of PCF [6]. Stapling is a very reliable method for pharyngeal repair. The closure is watertight with little to no contamination of the surgical field with oral and pharyngeal secretions [16, 17]. These facts explain better healing and lower incidence of PCF noticed in the present study.

Zhang et al. [18] reported that improper suture of the mucosa of the pharynx after total laryngectomy is one of the main reasons of PCF. Mechanical stapler closure is more precise and reliable method. Proper manual suturing requires skillful and meticulous surgical technique, in contrast stapler closure which is easy to perform and does not require long learning curve.

Lower fistula rates were achieved with stapler suturing compared to manual repair in many previous studies [6, 9, 18, 19]. In the large recent meta-analysis performed by Chiesa-Estomba et al. [20] in 2022, they compared stapler-assisted suture patients (242 patients) to manual suturing patients (380 patients). The incidence of PCF in the stapler group was 9.5% while in the manual group it was 23.4%. In contrast, Casasayas et al. [21] and Ahmed et al. [12] reported that the stapler did not affect the fistula rates.

Preoperative radiotherapy is a known risk factors for development of PCF [8, 9, 22]. In the present study, 21.4% of patients in the manual group, and 23.3% patients in the stapler group received preoperative radiotherapy, and the incidence of PCF was much lower in the stapler group denoting good outcomes in patients who received preoperative radiotherapy. Similarly, Galli et al. [23] performed a study to evaluate the stapler in salvage total laryngectomy and concluded that it decreased PCF rates.

Another beneficial advantage of stapler closure is shorter operative time. Time of pharyngeal closure using stapler in previous studies ranged from 2 to 5 min, while the manual closure time ranged from 20 to 50 min studies [12, 24]. In the current work, the mean stapler closure time was less than 2 min. Most patients who undergo total laryngectomy are elderly, with heavy smoking and multiple other medical comorbidities. Therefore, shorter operation time reduces the incidence of perioperative complications and morbidity [25]

The staples used for pharyngeal closure are titanium made, and do not react to magnetic fields and are safe to be used with the magnetic resonance imaging when required [26, 27].

Swallowing outcomes in the current study was better in the stapler group compared to the manual group. This may be due to single layer closure in the stapler group and double layer closure in the manual group, and consequently more incidence of cricopharyngeal spasm and strictures. Similar to our results, Bedrin et al. [9], in the most extensive stapler total laryngectomy series, published their 25- ear experience on the use of the linear stapler in 1415 patients. They concluded that the use of stapler techniques decreases surgical time, provides a watertight closure, and prevents field contamination. These outcomes are achieved with good deglutition outcomes.

Miles et al. [28] and Calli et al. [6] highlighted that application of the closed stapler techniques on an inappropriate patient may compromise the oncologic outcomes. Therefore, care should be taken to apply this technique on patients with strictly endolaryngeal tumors. In the current work, direct laryngoscopy was routinely performed after general anesthesia induction before starting total laryngectomy surgery. Tumors with extension to the tongue base, or hypopharynx were excluded from the study.

By adherence to the patient selection criteria in terms of tumor extension, our preliminary oncologic outcomes showed no significant difference in the overall 3-year survival rates, and disease specific survival rates between both stapler closure and manual closure groups.

A potential disadvantage of the staple is the higher cost. However, stapler technique provides shorter operative time and shorter hospital stay due to lower PCF rates and possible earlier oral feeding [9, 16, 24]. Consequently, by considering these indirect costs stapler technique is considered cost effective.

Limitations of the current study include small sample size and short follow-up period. Further studies including larger sample size and longer follow-up periods are warranted.

## Conclusion

The stapler is a reliable method for pharyngeal closure after total laryngectomy if the limits of its indications regarding the primary tumor are considered. Stapler closure decreases the incidence of PCF and decreases the surgical time. Good swallowing outcomes are achieved without compromising the oncological outcomes.

## Data Availability

The data supporting the findings of this study are available from the corresponding author upon reasonable request.
